# Repair of Segmental Load-Bearing Bone Defect by Autologous Mesenchymal Stem Cells and Plasma-Derived Fibrin Impregnated Ceramic Block Results in Early Recovery of Limb Function

**DOI:** 10.1155/2014/345910

**Published:** 2014-07-08

**Authors:** Min Hwei Ng, Suryasmi Duski, Kok Keong Tan, Mohd Reusmaazran Yusof, Kiat Cheong Low, Isa Mohamed Rose, Zahiah Mohamed, Aminuddin Bin Saim, Ruszymah Bt Hj Idrus

**Affiliations:** ^1^Tissue Engineering Centre, Universiti Kebangsaan Malaysia Medical Centre, Jalan Yaacob Latif, Bandar Tun Razak, Cheras, 56000 Kuala Lumpur, Malaysia; ^2^Department of Orthopaedics and Traumatology, Kuala Lumpur General Hospital, 50586 Kuala Lumpur, Malaysia; ^3^Orthopaedic, Traumatology and Spinal Surgery Consultant Clinic, Johor Specialist Hospital, 80100 Johor Bahru, Malaysia; ^4^Materials Technology Group (MTEG), Industrial Technology Division (BTI), Malaysian Nuclear Agency, Bangi, 43000 Kajang, Selangor, Malaysia; ^5^UKM Animal Resource Centre, Medical Faculty, Universiti Kebangsaan Malaysia, 50300 Kuala Lumpur, Malaysia; ^6^Department of Pathology, Universiti Kebangsaan Malaysia Medical Centre, 56000 Kuala Lumpur, Malaysia; ^7^Department of Radiology, Universiti Kebangsaan Malaysia Medical Centre, 56000 Kuala Lumpur, Malaysia; ^8^Ear, Nose & Throat Consultant Clinic, Ampang Puteri Specialist Hospital, 68000 Ampang, Malaysia; ^9^Department of Physiology, Medical Faculty, Universiti Kebangsaan Malaysia, 50300 Kuala Lumpur, Malaysia

## Abstract

Calcium phosphate-based bone substitutes have not been used to repair load-bearing bone defects due to their weak mechanical property. In this study, we reevaluated the functional outcomes of combining ceramic block with osteogenic-induced mesenchymal stem cells and platelet-rich plasma (TEB) to repair critical-sized segmental tibial defect. Comparisons were made with fresh marrow-impregnated ceramic block (MIC) and partially demineralized allogeneic bone block (ALLO). Six New Zealand White female rabbits were used in each study group and three rabbits with no implants were used as negative controls. By Day 90, 4/6 rabbits in TEB group and 2/6 in ALLO and MIC groups resumed normal gait pattern. Union was achieved significantly faster in TEB group with a radiological score of 4.50 ± 0.78 versus ALLO (1.06 ± 0.32), MIC (1.28 ± 0.24), and negative controls (0). Histologically, TEB group scored the highest percentage of new bone (82% ± 5.1%) compared to ALLO (5% ± 2.5%) and MIC (26% ± 5.2%). Biomechanically, TEB-treated tibiae achieved the highest compressive strength (43.50 ± 12.72 MPa) compared to those treated with ALLO (15.15 ± 3.57 MPa) and MIC (23.28 ± 6.14 MPa). In conclusion, TEB can repair critical-sized segmental load-bearing bone defects and restore limb function.

## 1. Introduction 

Bone nonunion secondary to either trauma or tumour resection has been known to pose great challenges in orthopaedic management. In our medical centre, we were presented with 631 cases of bone nonunion during the period 2005–2011, and tibial nonunion made up 45% of these cases. In adults and children, closed tibial shaft fractures are the most common long-bone fractures. Union of tibia is more difficult in view of the precarious blood supply and limited soft tissue coverage in this area [1, 2]. Such defects, which are unlikely to heal in the absence of secondary intervention, are commonly treated by means of osteotomy followed by bone distraction (the Ilizarov technique) or with the need for bone grafting [3, 4]. The Ilizarov technique involves a lengthy process and is often presented with pin track infections, pin loosening, and bone nonunion at the docking sites [5, 6]. Autologous bone grafting on the other hand, being the gold standard, is challenged by the limited availability of autogenous bone and the risk of donor site morbidity [7, 8]. These limitations are compounded in the elderly. Alternatively, segmental allogeneic bone grafts can be obtained from bone banking facilities [8]. Allogenic bone grafts are often subjected to demineralization to enhance their osteoinductive property. Demineralization of bone exposes the bone morphogenic proteins located within the bone matrix that play important roles in osteoinduction [9, 10]. Disadvantages of allogeneic bone grafting include risk of disease transmission, delayed vascular penetration, and delayed or incomplete graft incorporation [7, 8]. Furthermore, in countries where there are cultural taboos against tissue and organ donations, the supply of allogeneic bones is limited [11].

In view of the increased demands for bone grafts, alternative bone substitutes have been resorted. Amongst them, ceramic or calcium phosphate-based materials and bioglass appear to be the most favourable [12–16]. While most of these bone substitutes possess only osteoconductive property, they are now combined with osteoinductive materials such as growth factors, blood, bone marrow, and stem cells to create a new spectrum of bioactive bone substitutes [17–22]. Calcium phosphate-based bone substitutes have been in the market for decades and they come in various formats, powder, granules, self-polymerizing liquid, or miniblocks. Although their application as a single segmental block for repairing large segmental defects has been tested in animal models [12, 16, 23], their use in the clinic has yet to be reported. This is mainly due to their weak mechanical strength as they are expected to act at least initially as weight-bearing space fillers or struts in such applications.

Tissue engineering constructs incorporate osteoprogenitor cells and growth factors in a biodegradable scaffold to form a living bone substitute which is both osteoconductive and osteoinductive. We aim to fabricate a tissue-engineered bone construct from autologous or resorbable synthetic materials. This is to eliminate the risk of disease transmission and it will leave no residual foreign materials in the body upon complete bone regeneration.

Bone marrow is an ideal cell source to derive autologous osteoprogenitor cells. Studies have shown that bone marrow derived mesenchymal stem cells (MSCs) can be osteogenically induced to express specific osteogenic genes and mineralize within 2D and 3D matrices [24–26]. Liu et al. demonstrated that scaffolds seeded with osteogenically induced MSCs produced greater bone volume and density compared to scaffold seeded with noninduced MSCs [10]. Autologous platelet-rich plasma is a rich source of growth factors (GFs) such as platelet-derived growth factor- (PDGF-) AA, -BB, and -AB; transforming growth factor- (TGF-) beta 1 and -2; platelet-derived epidermal growth factor (PDEGF); platelet-derived angiogenesis factor (PDAF); insulin growth factor-1 (IGF-1); and platelet factor-4 (PF-4), which are known to stimulate bone regeneration [27]. The addition of autologous platelet-rich plasma or fibrin to the tissue- engineered bone constructs has been shown to promote osteogenic differentiation of MSCs* in vitro* [22, 27, 28] and further enhanced osteogenesis* in vivo *[29, 30].

Calcium phosphate-based ceramic is bioresorbable, mimics the content of a natural bone, and can be chemically synthesized. The tricalcium phosphate (TCP) form of ceramic is highly degradable while hydroxyapatite (HA) is structurally stronger but resorbs slowly [31]. Studies have shown that TCP/HA, as a composite, encouraged greater bone matrix synthesis compared to hydroxyapatite (HA) or tricalcium phosphate (TCP) alone [29, 32]. In this study, tissue-engineered bone constructs composed of autologous osteogenic-induced mesenchymal stem cells and plasma-derived fibrin seeded onto TCP/HA scaffolds were evaluated.

The study was conducted as a proof of concept for the use of autologous tissue-engineered bone construct as an alternative bone substitute for segmental defect of load-bearing bone in animal models. Although similar combinations have been tested in animal models, limb function was not evaluated. To further test the feasibility of using such combination in place of current popular bone substitutes in the operating theatres, partially demineralized allograft bone and fresh autologous marrow-impregnated ceramics were tested in parallel [9, 10, 20, 32, 33].

## 2. Materials and Methods 

### 2.1. Animal Models

**Figure 1:**
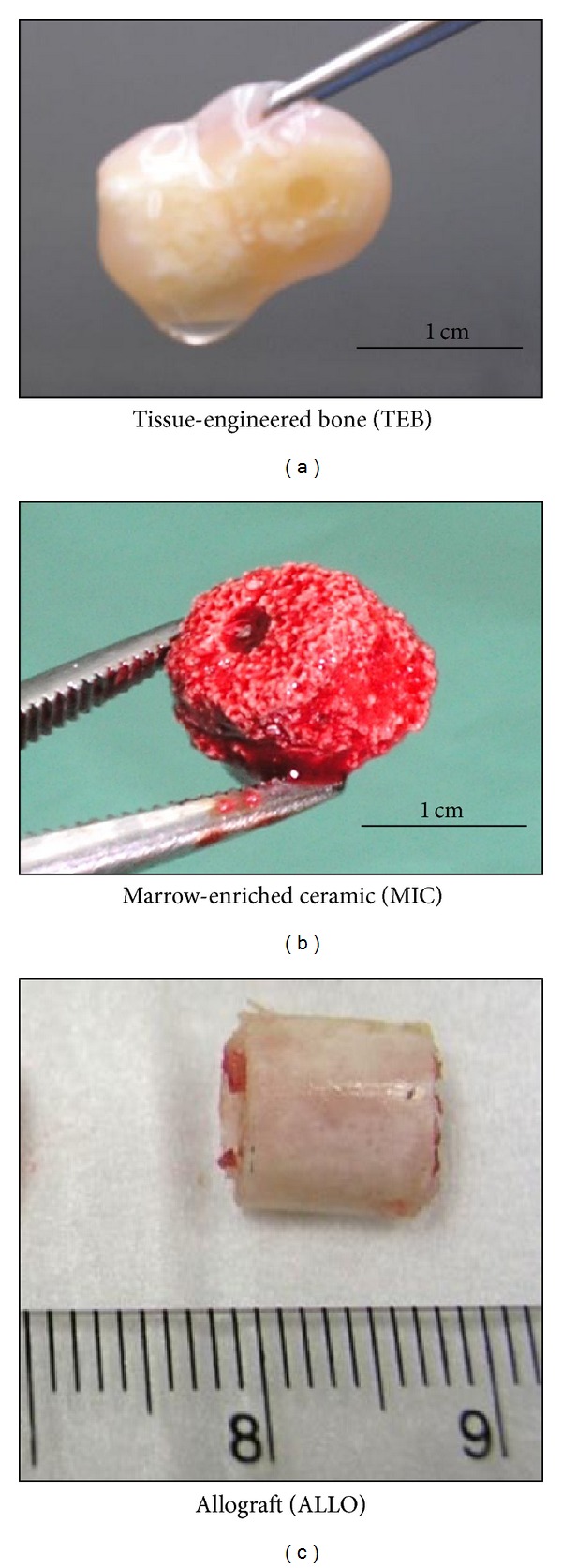
Gross appearance of implants used in the three treatment groups.

The study has been approved by the Institution Research and Ethics Committee (Code no. UKM 1.5.3.5/244/PPP2). Female New Zealand White rabbits aged approximately 6 months and weighed approximately 2.5 kilograms were used as animal models. Each rabbit was subjected to a one-centimetre segmental defect at the midshaft of the left tibia and was randomly assigned for treatment with one of the three bone substitutes, namely, autologous tissue-engineered bone construct (TEB; *n* = 6), partially demineralized tubular tibia allograft (ALLO; *n* = 6), or fresh autologous marrow-impregnated ceramic (MIC; *n* = 6) (Figure 1). Three rabbits without bone substitutes were used as negative controls (CTRL; *n* = 3).

### 2.2. Preparation of Autologous Tissue-Engineered Bone Construct (TEB)

TEB was comprised of autologous MSCs seeded onto porous ceramic scaffold enriched with autologous plasma-derived fibrin. MSCs were derived from rabbit bone marrow and expanded* in vitro*. Fibrin was derived from the rabbit whole blood. Cell-fibrin mixture was then seeded onto a cylindrical ceramic scaffold followed by induction in osteogenic medium. Detailed procedures are as follows.

#### 2.2.1. Autologous Rabbit Bone Marrow and Blood Harvesting

Rabbits were anaesthetized by intravenous administration of a drug mixture (Xylazine, Ketamine, and Zoletil). Right iliac crest region of the rabbit was shaved, cleaned, and draped. One centimetre skin incision was made over the iliac crest. Five millilitres of rabbit bone marrow was harvested percutaneously from the rabbit right iliac crest using an 18 G needle. At the same time, 5 mL of blood was aspirated from the lateral ear vein into a sodium citrate tube.

#### 2.2.2. Plasma-Derived Fibrin Preparation

The collected blood was centrifuged at 3000 rpm for 5 minutes. After centrifugation, the plasma layer (top yellowish layer) was transferred to a new tube and stored at −20°C before use.

#### 2.2.3. *In Vitro* Cell Expansion

Mononuclear cells were isolated from the aspirated bone marrow via gradient centrifugation over a Ficoll-Paque (GE healthcare, USA) layer. The isolated cells were suspended in alpha-minimum essential media (*α*-MEM; Invitrogen, USA), supplemented with 10% fetal bovine serum (Invitrogen, USA), and plated onto a 9.6 cm^2^ culture plate. All cultures were incubated at 37°C in a humidified atmosphere of 5% CO_2_. Medium was changed twice a week. Upon cell confluence, cells were detached by treatment with 0.05% trypsin-EDTA solution. Cell count and cell viability were assessed using the trypan blue dye-exclusion method. Subsequently, cells were subcultured until Passage 3 at a standard density of 5000 cells/cm^2^.

#### 2.2.4. Ceramic Scaffold Preparation

Porous ceramic blocks composed of 80% *β*-tricalcium phosphate and 20% hydroxyapatite were obtained commercially (Surgiwear, India). They are chemically synthesized to produce a nominal surface porosity of 50% and pore sizes ranging from 200 to 500 *μ*m. In the laboratory, they were molded with a burr into hollow cylinders of 1 cm in length with an outer diameter of 0.7 cm and an inner diameter of 0.3 cm. They were then individually packed, sterilized by gamma-irradiation (30 kGy), and stored at room temperature before use.

#### 2.2.5. *In Vitro* TEB Preparation

Approximately 100 million cells from Passage 3 cultures were suspended in 1 mL of autologous platelet-rich plasma and seeded on each cylindrical ceramic scaffold. Calcium chloride was added to the cell-seeded scaffold in order to initiate fibrin polymerization. The cell + fibrin seeded scaffold was then immersed in osteogenic differentiation medium (*α*-MEM with 10% fetal bovine serum, 10–7 M dexamethasone, 0.05 mg/mL ascorbate-2-phosphate, and 10 mM beta-glycerophosphate) with continuous agitation (dynamic culture) on a shaker (Kiddy See Saw KSS-01, Toylab, Republic of Korea) for one week. The method of seeding was previously validated for optimal seeding efficiency and cell viability [34].

### 2.3. Preparation of Marrow-Enriched Ceramic (MIC)

On the day of surgery, 5 mL of bone marrow was aspirated from the rabbit right iliac crest and transferred to a sterile container. The sterile cylindrical ceramic cylinder (prepared as stated in Section 2.2.4) was then placed fully immersed in the aspirated bone marrow for at least 30 minutes before implantation.

### 2.4. Preparation of Partially Demineralized Allograft (ALLO)

Partially demineralized allogeneic bone grafts were prepared from tibiae of six-month old rabbits, according to a published protocol [10]. Briefly, bone grafts were segmented from the diaphysis of tibiae, followed by the removal of periosteum and any adhering soft tissues. The allografts were then snap frozen at −80°C for 24 hours, cut into one-centimetre long segments, and soaked in The sterile cylindrical ceramic cylinder 100% ethanol for another 24 hours. This was followed by soaking of the allografts in a 0.6 N hydrochloric acid for 15 minutes (50 mL HCl per gram of bone). They were then air dried, individually packed, gamma irradiated (30 kGy), and stored at −80°C before use.

### 2.5. Bone Substitute Implantation and Fixation

Under general anaesthesia, left tibial region of the rabbit was shaved and cleaned. A custom-made external fixator was placed over the lateral side of the tibial midshaft. Three 1.6 mm K wires were driven through the fixator rod and through both cortices of the tibia flanking two sides of the tibial defect (Figure 1). A longitudinal skin incision was then made over the lateral aspect of the tibia. Muscles and vessels were retracted and the midshaft of the tibia was exposed. A 1 cm segmental defect was made over the midshaft of the rabbit tibia using an electric burr, and periosteum surrounding the defect was completely removed. Another longitudinal skin incision was made over the medial aspect to the patellar tendon. A 0.8 mm K-wire was driven via the patellar through the intramedullary canal, through the respective study implant, and then lodged into the distal subchondral bone. Wounds were closed using 3.0 nylon sutures.

### 2.6. Postoperative Care

Postsurgical rabbits were housed individually in a 3 × 2 × 2 feet cage, with food and water ad libitum. Intramuscular administrations of antibiotics (cyclopropyl; Bayer AG) and analgesic (meloxicam; Boehringer Ingelheim) were given for 5 days after surgery. The rabbits were regularly monitored for signs of infection and instability of fixations. Dressing was changed whenever necessary.

### 2.7. Radiographic Analysis

Bone union rate was monitored by a series of anteroposterior and lateral radiographs of the tibia at Day 0 (immediate after operation), Day 7, Day 21, Day 60, and Day 90 (three months). Radiographs were taken using a standardized setting throughout the study. Degree of union for each rabbit was graded at Day 90, using a numerical score based on criteria modified from Salkeld et al., (Table 1) [35]. Three researchers blinded to the study performed the grading independently.

### 2.8. Gait Analysis

Gait analysis was performed on a pressure recording mat to assess tibial function after bone repair. Gait pattern was recorded prior to the surgery and at Day 90 after operation. Analysis of the contact pressure and contact area was performed on the MatScan Research version 5.72 software.

### 2.9. Biomechanical Analysis

At Day 90, rabbit was sedated via intravenous ketamine injection followed by intracardiac pentobarbital injection. Specimens were then harvested 2.5 cm distally and proximally to the implant using a sharp diamond cutter. Both ends of the harvested specimens were mounted in fast hardening resin (SeriFix Resin). Uniaxial compression test was performed using a servohydraulic compression-testing machine (Instron Compression Test Systems model 8874, MA, USA) equipped with a 25 kN load cell. The specimens were subjected to compression at a constant displacement rate of 1 mm/minute of crosshead velocity until failure. Compressive stress and strain were calculated and plotted. Stress value at the point of yield (load-to-failure) was determined. In an effort to reduce errors associated with geometric differences among the animals, the ratio of compressive strength of the experimental tibia to the contralateral tibia was presented.

### 2.10. Histological and Histomorphometric Analyses

After biomechanical testing, specimens at the bone-implant interphase were fixed with 10% buffered formalin overnight. The fixed specimens were subjected to decalcification in EDTA-saturated 4% HCl for one week followed by routine tissue processing and paraffin embedding. Longitudinal sections of 5 *μ*m were prepared using a microtome. The tissue sections were dewaxed, rehydrated, and stained with hematoxylin and eosin or Alizarin red. The slides were evaluated by a qualified pathologist blinded to the study. Evaluation criteria included amount of new bone formation and residual implant and degree of fibrosis and inflammation. In addition, quantification of the mean percentage of residual implant, osteoid, and mature bone was performed on four representative Alizarin Red-stained sections for each group, using Image-Pro Plus version 6.2.1 (Media Cybernetics, Bethesda, USA). Mean percentage was calculated with respect to the total area covered by materials and tissues on each section.

### 2.11. Statistical Analysis

Quantitative data is expressed as mean ± standard error of mean (SEM). Student's *t*-test was performed to compare difference of mean between 2 groups using PASW statistics 18 (SPSS Inc.). *P* value of less than 0.05 is considered statistically significant.

## 3. Results

### 3.1. Radiological Findings

**Figure 2:**
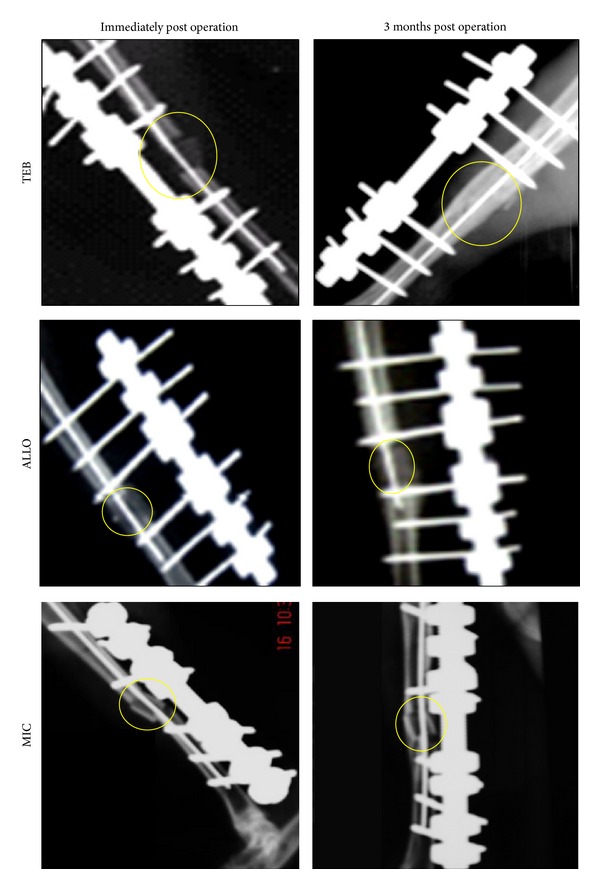
Radiological changes seen in the three test groups immediately: Day 21, Day 60, and Day 90 after operation. TEB: defect bridged by uniform new bone, cut ends of cortex no longer distinguishable, graft no longer distinguishable. MIC: a slight increase in radiodensity surrounding and distinguishable from the graft (callus formation) with no bridging of cortex. ALLO: a slight increase in radiodensity surrounding and distinguishable from the graft bridging of one cortex with new bone formation.

Radiographs of the test and control tibiae are shown in Figure 2 and the score results are summarized in Table 2. No observable change was noted up to Day 7 across all groups. However, from Day 21 onwards, TEB score was the highest in mean radiological grade (*P* < 0.05). By Day 90, all rabbits in TEB showed bridging of cortices by dense radiopaque new bone and of which two achieved complete union on all sides. One rabbit in ALLO and two in MIC achieved bridging of cortices.

### 3.2. Gait Analysis

**Figure 3:**
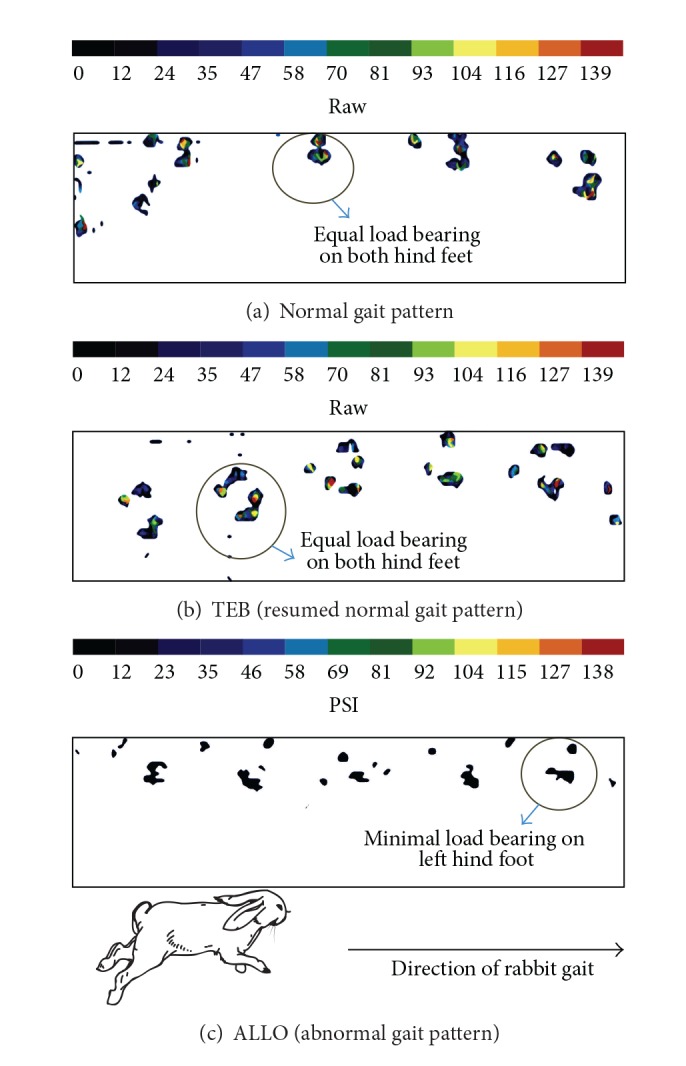
Gait pattern recorded with TechScan software. (a) Hopping pattern of a normal rabbit with even pressure on both hind limbs. (b) A near normal hopping pattern of a rabbit from TEB group at Day 90 after operation. (c) A distorted gait pattern of a rabbit from ALLO group showing unloading of the experimental limb (colour bars indicate pressure in PSI).

Example of a normal, near normal, and abnormal rabbit gait pattern is shown in Figure 3. At day 90, two out of the six rabbits in ALLO and MIC exhibited a normal gait pattern, while four out of six rabbits from TEB resumed a normal gait pattern. Most of the abnormal gait patterns were due to overbearing of weight and pressure on the nonoperated contralateral hind limb, nonloading or partial loading of the operated limb, or dragging of the operated limb. The restoration of normal gait pattern was taken as an indication for the restoration of function of the operated limb and the absence of implant complications.

### 3.3. Gross Evaluation

**Figure 4:**
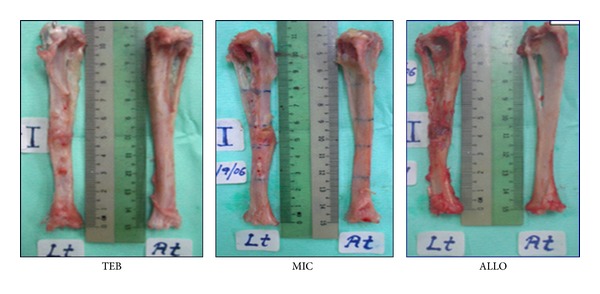
Gross appearance of postimplanted experimental tibia compared with contralateral controls. All groups maintained equal length between test and contralateral tibiae. TEB showed good continuity of cortices along the bone-implant-bone interphases. Implant material for ALLO and MIC groups are still visible. Lt: implanted left tibia; Rt: normal right tibia.

Regardless of partial or complete union, bone lengths of the treated limbs were maintained in all treatment groups. Three rabbits in TEB achieved complete union showing good continuity of cortices along the bone-implant-bone interphases while the other three showed partial union. In this group, graft could no longer be distinguished from the surrounding bone (Figure 4). In ALLO group, partial union was seen in four rabbits and nonunion in two. In these nonunion rabbits, residual allografts were still visible and soft tissues were found enveloping the allografts. In MIC group, partial union was seen in three of the rabbits and nonunion in other three. In these nonunion rabbits, substantial amount of residual ceramic surrounded by soft tissues were noted. No bone union was achieved in the negative control group.

### 3.4. Biomechanical Findings

Rabbits with partial or complete union were subjected to biomechanical testing. Results of the biomechanical tests are summarized in Table 3. TEB-treated tibiae exhibited the highest compressive strength (*P* < 0.05) while ALLO and MIC-treated tibiae (*P* > 0.05) were similar in strength. TEB-treated tibiae achieved between 15 and 49% of the strength of the contralateral tibiae.

### 3.5. Histological and Histomorphometric Findings

**Figure 5:**
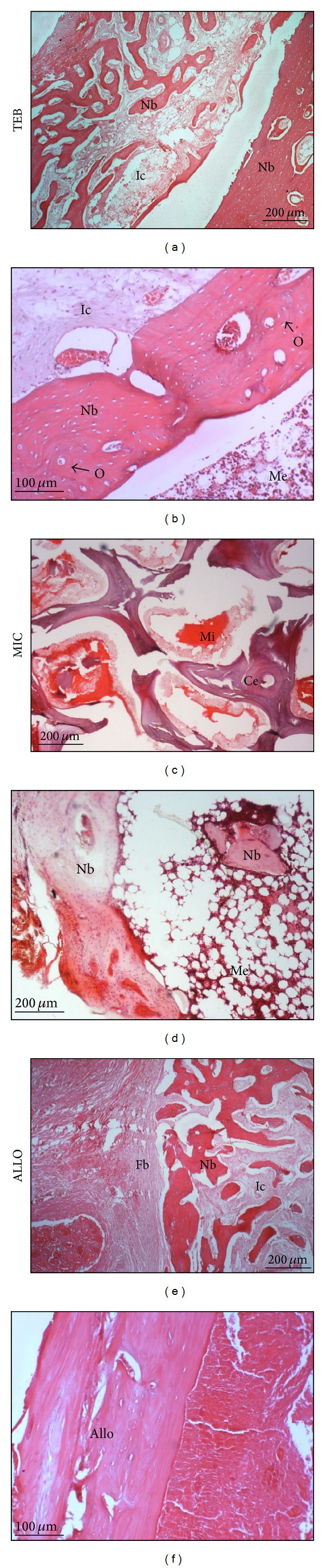
Histological sections from the middle segment of the implants three months after implantation (H&E). (a) Abundant new bones were found in TEB. The section reveals new bones (Nb) forming a trabecular network amidst infiltrated cells (Ic) while new compact bone (Nb) was found at the right periphery (40x). (b) Here, the peripheral bone appeared more mature with lamellar and osteon features (O) adjacent to the well-formed intramedullary canal filled with marrow element (Me) (100x). (c) Residual ceramic (Ce) was noted in MIC. Mineral deposits (Mi) (stained red) were seen around the ceramic (40x). (d) The section reveals new bones (Nb) that are undergoing mineralization amidst infiltrated marrow element (Me) (40x). (e) Significant fibrous tissues (Fb) were noted in ALLO. The section reveals new bones (Nb) forming a trabecular network amidst infiltrated cells (Ic) (40x). (f) An intact allograft bone (Allo) (100x).

Figure 5 shows H&E stained sections of specimens taken from the middle segment of the implants three months after implantation. In TEB group, abundant new bone was found forming a trabecular network within the medullary cavity. Advance bone remodeling occurred in TEB group as evidenced by the presence of mature cortical bone at the periphery. The cortical bone was found along the entire gap of the bone defect bridging to the adjacent native bone. Intramedullary canal was maintained in which marrow elements flowed through. Almost the entire of the ceramic scaffold of the TEB had resorbed. Occasional scattered ceramic granules were noted. In the MIC group, substantial amount of the ceramic scaffolds remained. Mineral deposits were found accumulated around these degrading ceramic, and possibly the product of ceramic degradation was seen around the ceramic (Figure 5(c)). New bones found in MIC group appeared less mature. In ALLO group, significant fibrous tissues (Fb) were noted. Trabecular bone was actively being laid down next to these fibrous tissues. Most part of the allograft bone remained intact and devoid of any bone growth or remodeling activities. These bones were marked by empty lacunae with no resident cells (Figure 5(f)).

Histologically, TEB and MIC groups showed comparable amount of new bone. Fibrosis and inflammatory cells were occasionally noted in MIC and ALLO groups, but not in the TEB group.

**Figure 6:**
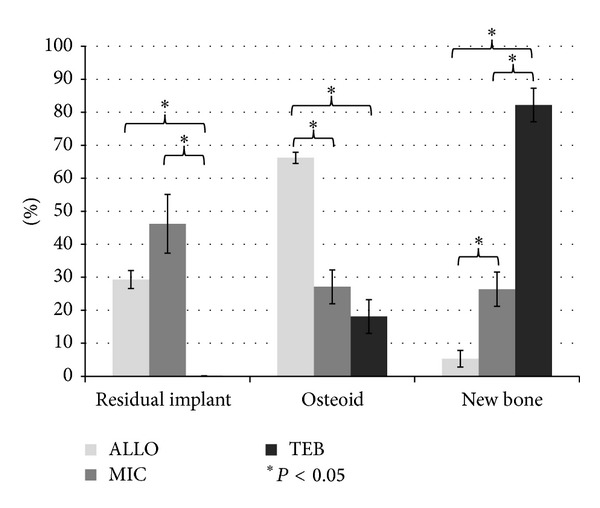
Histogram showing the mean percentage of residual implant, osteoid, and mature new bone determined in representative sections of TEB, MIC, and ALLO specimens.

Figure 6 shows a histogram of the mean percentage of residual implant, osteoid, and mature new bone from the implants. Mean percentages of residual implant materials for ALLO, MIC, and TEB groups were 29% ± 2.7%, 46% ± 8.9%, and 0.1% ± 0.02%, respectively. Mean percentages of osteoid or precursor bone for ALLO, MIC, and TEB groups were 66% ± 1.7%, 27% ± 5.1%, and 18% ± 5.1%, respectively, and mean percentages of new bone were 5% ± 2.5%, 26% ± 5.2%, and 82% ± 5.1%, respectively.

## 4. Discussions 

Combinations of varying scaffold materials, cell sources, and growth factors have been used to construct tissue-engineered bone. Ceramic remains the most popular type of scaffold material due to its bioresorbablity and osteoconductive property. The addition of biological factors has been proven to provide osteoinductive property to the implants [17–22]. However, the mechanism by which these biologics act in the bone regeneration and remodeling process remains vague.

The combination of ceramic, cells, and fibrin as a bone substitute is not new. However, to date, only one study reported the use of such combination in segmental bone repair [35]. Moreover, due to the high variability in the configuration and preparation of the ceramic scaffolds, cells, or fibrin, all translational laboratories will need to develop, optimize, and test their own products. The tissue-engineered bone used in this study has been optimized via a series of developmental studies* in vitro* and* in vivo *[25, 26, 32, 34].

The use of such combination for segmental long bone defect is met with specific challenges. The choice of fixation methods greatly impacts the repair outcome [36]. A rigid fixation is required to achieve good alignment and stability of the implants. Postoperative care for such animal models is extremely important. Ultimately, the aim of the repair is to achieve early union and weight bear. Early mobility will reduce the risk of developing bed sores and pressure ulcers, especially important for the elderly as they are often accompanied by poor blood circulation and diabetes.

The primary strength of this study is the introduction of an objective method to evaluate limb function. The restoration of limb function after bone repair is determined using gait analysis via foot pressure recordings. It is also an important indication that the implants do not cause complications to other functions of the limb such as soft tissue inflammation. In addition, the testing of the combination was performed in parallel with two other popular bone substitutes.

Our radiological findings showed that TEB group had accelerated bone healing and union when compared with ALLO and MIC groups. Increased density at the implant site and early fusion of cortical bone could be seen in TEB group as early as Day 21 after implantation. There was no significant difference between ALLO and MIC groups in terms of rate of union. The accelerated rate of healing in TEB group resulted in four out of six rabbits' return to normal gait within 3 months after surgery. This may potentially translate into early removal of fixator and early full weight bearing for patients.

Grossly, union was assessed by palpation and the naked eye. Union was perceived as any bridging of bone gap with hard tissues. Complete union was reported when all 4 sides of the cortex were bridged. However, the quality of union had to be further assessed by biomechanical testing and histology. In our findings, gross union concurred with radiological union.

In this study, we aimed to mimic load transfer to the tibia to determine if the repaired bone could indeed withstand the load from the rabbit after the removal of the fixators. Hence, compression test was performed. Although torsional or 3-point bending tests could be more appropriate to study the strength of bone-implant interface, it could not be performed due to the lacking of appropriate jig in our facilities. Instead, quality of union could be inferred based on the fracture site at the point of yield during compression testing. When fracture occurs at the bone-implant interphase, union is considered poor and the implant is relatively stronger. On the other hand, when fracture occurs at the implant site, union is relatively stronger than the implant and the compressive strength recorded reflects the strength of the implant. In TEB and MIC groups, fracture occurred at the implant site. This is suggestive of the relatively strong bone-implant integration. TEB group scored a higher compressive strength than MIC group and this implies that TEB formed greater amount of bone than MIC. On the other hand, fracture occurred at the bone-implant interphase in the ALLO group, which implies a relatively strong implant but poor bone integration.

Histologically, complete remodeling at the bone defect site was seen only in TEB, producing well-formed intramedullary canal filled with marrow (Figure 5(b)). Our results are consistent with a recent report by Nair et al. whereby their approach using cells and plasma-enriched cylindrical hollow hydroxyapatite produced mature bone with the regeneration of marrow cavity [23]. Previous approaches using packed-filled granular or solid ceramic block achieved solid fusion without the formation of intramedullary canal. The approach may block the intramedullary blood circulation, increase intraosseous pressure and result in altered bone physiology in the long term [37]. The logic behind the design of a hollow and porous cylindrical scaffold in our study is to allow fresh marrow to flow through the scaffold so that fresh nutrient from the bone marrow and waste products from the seeded cells can easily diffuse through the scaffold. A hollow scaffold also permitted the use of intramedullary nail for enhanced fixation and alignment. As compared with ceramic particulates, ceramic cylinder mimics the shape and size of the segmental bone defect and acts as a temporary structural support that bridges the bone gap.

TEB achieved comparable if not superior outcome in all aspects of evaluation. We have shown in our findings that tissue engineering strategy is more effective than simple delivery of crude bone marrow with ceramic scaffolds. The enhanced osteogenic property of the tissue-engineered constructs can be attributed to the presence of a large number of osteogenically induced stem cells and growth factor-rich plasma-derived fibrin. In the study, osteogenically induced MSCs were incorporated into TEB. We have shown in our previous work that osteogenically induced MSCs readily produced the required bone matrix proteins. We have previously found that fibrin derived from plasma enhanced matrix formation in a 3D construct [29, 34].

While the combination of ceramic with MSCs and plasma-derived fibrin may not be novel, a completely autologous approach has not been reported [19, 22, 23, 38]. Tissue engineering strategy allows a complete autologous approach. Autologous serum can also be derived from patients instead of fetal bovine serum for culturing the autologous MSCs.

This will eliminate the risk of disease transmission and tissue rejection. With time, the scaffold will degrade and be completely replaced by the autologous new bone. We postulate that the calcium and phosphate released from the degradation of the ceramic scaffold have in turn been assimilated into the new bone.

In the study, tissue-engineered bone was successfully constructed within 3 weeks from time of bone marrow aspiration. In our experience, sufficient MSCs can be generated through* in vitro* cell expansion in 3 weeks. The delayed supply of autologous TEB may be seen as one of the limitations of TEB strategies as compared to other off-the-shelf products. However, the delayed in supply of autologous TEB does not pose a real problem to the treatment for nonunion or delayed union. Even for fracture cases resulting from trauma, often time is required for patients to stabilize before bone grafting can be performed. The real barrier to translation for autologous TEB would be the high cost of production. Hence, the future trend is to resort to allogeneic, off-the-shelf products. A totally allogeneic approach is now possible because MSCs derived from certain sources such as umbilical cord are thought to be immunoprivileged; that is, they do not evoke an immune reaction. Similarly, allogeneic plasma-derived fibrin will not evoke any immune reactions as it is devoid of cells.

To better simulate the clinical scenario, we suggest that treatment should be done in our future studies in a nonunion model instead of an immediate implantation upon the creation of the bone defect. Next, larger animal models should be used to test the efficacy of larger tissue-engineered bone constructs. In addition to compression testing, torsion should also be performed to better determine the degree of scaffold integration with the host bone.

## 5. Conclusions 

Tissue-engineered bone construct comprised of segmental ceramic block impregnated with osteogenically-induced autologous MSCs and plasma-derived fibrin can be an alternative to bone allograft for repairing critical size load-bearing segmental bone defect, early weight loading, and recovery of limb functions. A completely autologous concept can be implemented via this approach. Nonetheless, the more commercially viable strategy into the clinic is the use of immune privileged MSCs and plasma-derived fibrin from allogeneic source.

## Figures and Tables

**Table 1:** Radiological grading scale.

Description	Grade
No change from immediate postoperative appearance	0
A slight increase in radiodensity distinguishable from the graft	1
Recognizable increase in radiodensity, bridging one cortex with new-bone formation to the graft	2
Bridging of at least one cortex with material of nonuniform radiodensity and early incorporation of the graft suggested by obscurity of graft borders	3
Defect bridged on both medial and lateral sides with bone of uniform radiodensity; visible cut ends of the cortex; not-easy-to-differentiate graft and new bone	4
Same as grade 3, with at least one of four cortices obscured by new bone	5
Defect bridged by uniform new bone; cut ends of cortex no longer distinguishable; graft no longer visible	6

Source: Salkeld et al., 2001 [35].

**Table 2:** Mean and range of radiological scoring for all test groups by three independent investigators.

Group	Day 7 Mean ± SEM (range)	Day 21 Mean ± SEM (range)	Day 60 Mean ± SEM (range)	Day 90 Mean ± SEM (range)
TEB	0 (0)	1.5 ± 0.52 (0–4)	2.25 ± 0.61 (1–6)	4.50 ± 0.78 (1–6)
MIC	0 (0)	0.30 ± 0.21 (0–2)	0.8 ± 0.24 (0–2)	1.28 ± 0.24 (0–3)
ALLO	0 (0)	0.33 ± 0.21 (0–2)	0.83 ± 0.30 (0–3)	1.06 ± 0.32 (0–3)

**Table 3:** Mean compressive strength ratio across the 3 test groups.

Group	Mean compressive strength (MPa)	Mean relative compressive strength (ratio to the contralateral tibia)
TEB (*n* = 6)	43.50 ± 12.72	0.28 ± 0.06
MIC (*n* = 3)	23.28 ± 6.14	0.15 ± 0.01
ALLO (*n* = 4)	15.15 ± 3.57	0.15 ± 0.04
